# Monitoring Cell Adhesion on Polycaprolactone–Chitosan Films with Varying Blend Ratios by Quartz Crystal Microbalance with Dissipation

**DOI:** 10.1021/acsomega.3c01055

**Published:** 2023-05-05

**Authors:** Ayşe Buse Özdabak Sert, Eva Bittrich, Petra Uhlmann, Fatma Nese Kok, Abdulhalim Kılıç

**Affiliations:** † Department of Molecular Biology and Genetics, Istanbul Technical University, 34469 Istanbul, Turkey; ‡ Leibniz-Institut für Polymerforschung Dresden e.V., 01069 Dresden, Germany

## Abstract

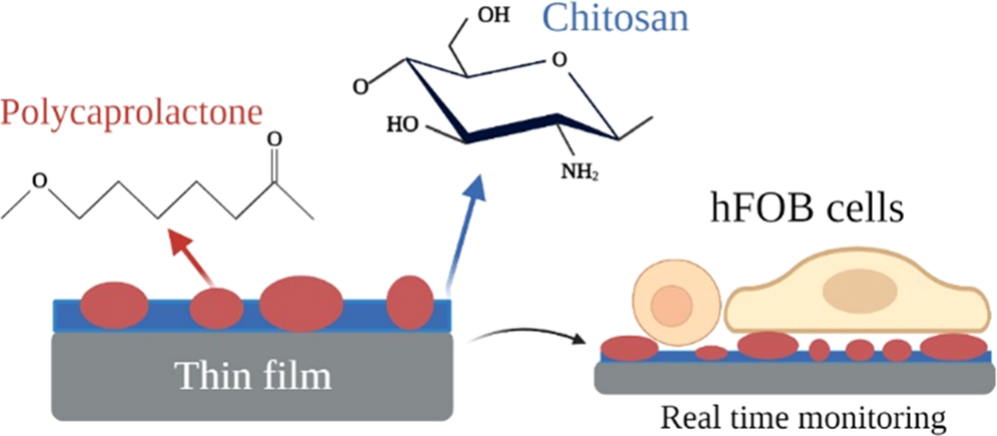

A detailed understanding of the cell adhesion on polymeric surfaces is required to improve the performance of biomaterials. Quartz crystal microbalance with dissipation (QCM-D) as a surface-sensitive technique has the advantage of label-free and real-time monitoring of the cell–polymer interface, providing distinct signal patterns for cell–polymer interactions. In this study, QCM-D was used to monitor human fetal osteoblastic (hFOB) cell adhesion onto polycaprolactone (PCL) and chitosan (CH) homopolymer films as well as their blend films (75:25 and 25:75). Complementary cell culture assays were performed to verify the findings of QCM-D. The thin polymer films were successfully prepared by spin-coating, and relevant properties, *i.e.*, surface morphology, ζ-potential, wettability, film swelling, and fibrinogen adsorption, were characterized. The adsorbed amount of fibrinogen decreased with an increasing percentage of chitosan in the films, which predominantly showed an inverse correlation with surface hydrophilicity. Similarly, the initial cell sedimentation after 1 h resulted in lesser cell deposition as the chitosan ratio increased in the film. Furthermore, the QCM-D signal patterns, which were measured on the homopolymer and blend films during the first 18 h of cell adhesion, also showed an influence of the different interfacial properties. Cells fully spread on pure PCL films and had elongated morphologies as monitored by fluorescence microscopy and scanning electron microscopy (SEM). Corresponding QCM-D signals showed the highest frequency drop and the highest dissipation. Blend films supported cell adhesion but with lower dissipation values than for the PCL film. This could be the result of a higher rigidity of the cell–blend interface because the cells do not pass to the next stages of spreading after secretion of their extracellular matrix (ECM) proteins. Variations in the QCM-D data, which were obtained at the blend films, could be attributed to differences in the morphology of the films. Pure chitosan films showed limited cell adhesion accompanied by low frequency drop and low dissipation.

## Introduction

Protein adsorption and subsequent cell adhesion are crucial for the *in vivo* performance of biomaterials. Physicochemical properties such as topography, surface charge, and stiffness have a great impact on the cell attachment process; therefore, their effect on cell attachment should be known to tailor the performance of prospective implants.^1^ Interfacial cell/protein–material interactions are complex and should be well understood and well designed in order to fulfill specific application requirements.^2^ Suppression of protein adsorption and cell adhesion is desired, for instance, for a biomaterial that will be used as a vascular implant or catheters. This can be achieved by the rational design and control of surface properties or by modification with biological molecules to prevent or enhance cell adhesion.^3^

Polycaprolactone (PCL) is a well-known and widely used synthetic and biodegradable polymer. Usage of PCL as a biomaterial is limited due to its hydrophobicity. For an improvement of the properties, various techniques have been reported to tailor the composition and/or surface properties. Another strategy to overcome the mentioned limitations is to modify or blend PCL with natural hydrophilic polymers. For example, PCL is modified with galactose to reduce inflammation,^4^ blended with gelatin to increase mineralization,^5^ or blended with chitosan to induce antibacterial properties.^6^ Blends of PCL with chitosan are used in various applications such as wound healing,^7^ cell migration,^8^ or bone tissue engineering.^9^ In addition, previous studies showed that while cell attachment and proliferation on pure PCL were low, addition of natural polymers led to higher cell adhesion.^10,11^ Therefore, blending of PCL with chitosan is used to improve the performance of PCL as a biomaterial; however, detailed studies considering the interactions at the cell–biomaterial interface are limited. For cell adhesion studies on PCL and chitosan polymers, researchers generally use end-point assays, in which fixing, labeling, and visualization steps of the cells under the microscope are required.^12−14^ Even in the case when two different cells look microscopically similar, in reality, the interfacial interactions, occurring in nanometer scales between the cell and the surface, may differ remarkably and thus cannot be distinguished by those types of assays.^15,16^ Further, these interfacial interactions are often difficult to probe directly.^17^ At this point, quartz crystal microbalance with dissipation (QCM-D) is a powerful technique for real-time monitoring of cell–surface interactions at the nanoscale as it has a maximum sensing depth of around 250 nm.^18^ Furthermore, QCM-D is label-free and thus provides the characteristic data for specific interactions at the cell–material interface in a noninvasive manner,^19^ which enables new insights into the cell adhesion behavior. This was shown for solvated homogeneous as well as heterogeneous interfaces.^20^

There are several studies that monitor cell adhesion by QCM-D. Many of the previous works monitored cell attachment and spreading in real time on bare sensor surfaces,^2^ sensors modified with cell attractive molecules,^17^ rigid surface coatings,^15,21^ self-assembled monolayers with different surface potentials,^1^ or supported lipid bilayers.^22,23^ Besides cell adhesion studies, QCM-D could be employed to follow layer-by-layer assembly of polymers,^24,25^ to examine polymer swelling at various conditions,^26^ and protein adsorption kinetics at different surfaces.^27−30^ Yet, the number of studies investigating cell adhesion on polymeric surfaces by QCM-D is limited.^31^

The initial contact during cell adhesion onto a surface establishes through nonspecific interactions. As the cells interact more intensively with the surface, they flatten and attach *via* their integrins to make protein-mediated specific interactions in time.^32,33^ QCM-D records changes in frequency (Δ*f*) and changes in energy dissipation (Δ*D*), while the real-time monitoring of Δ*f* and Δ*D* signals gives insights into the cell adhesion kinetics. To follow cell adhesion, changes in frequency can be related to the adsorbed mass and surface coverage of the cells^14^ and secretion of microexudates.^34^ The dissipation signal is related to viscoelasticity changes,^35,36^ attributed to a combination of many different events such as changes in mechanical properties and rearrangements of the cytoskeleton.^17^ However, for cell adhesion studies followed by QCM-D, already various and often unique frequency/dissipation responses for different types of cells have been identified on different types of surfaces.^2^ The complexity of the cell adhesion process complicates the interpretation of the magnitude and direction of the changing signals during this process.^37^ In addition to this, when cell adhesion is monitored on nonrigid films, like swollen polymer surfaces, mechanically trapped water in the system should additionally be taken into consideration.^37^

The purpose of this study is to investigate how the chemistry and morphology of PCL and chitosan films and their blends influence cell adhesion by means of QCM-D. Within the study, the adhesion of human fetal osteoblastic bone (hFOB) cells on various thin films was monitored in real time and label-free. Complementary cell culture assays are presented to verify the findings of QCM-D. For a detailed understanding of the cell–surface interactions, a comprehensive surface analysis of the used homopolymer and blend films was performed before adsorption. The surface morphology was investigated by atomic force microscopy (AFM), chemical analysis was performed by Fourier transform infrared spectroscopy (FTIR), and wettability was measured using dynamic contact angles. Dry and swollen thicknesses were obtained by spectroscopic ellipsometry. Fibrinogen adsorption was probed by QCM-D and correlated to cell adhesion. Revealing the dynamic adhesion behavior of the cells on those films will provide valuable information for the design of biomaterials for different applications.

## Results and Discussion

### Thin-Film Characterization

Attenuated total reflectance-FTIR (ATR-FTIR) spectra prove that both of the polymers were successfully coated onto the surface (Figure S1). PCL has a characteristic peak at 1725 cm^–1^, which belongs to the carbonyl stretching vibration visible in bare PCL and all blend coatings.^38^ Chitosan has characteristic peaks at 1645 cm^–1^ for the amide I stretching vibration, 1584 cm^–1^ for the N–H bending vibration of amine, and around 3290 cm^–1^ for the N–H stretching vibration of amine.^39^ These absorption bands can be clearly seen in the infrared (IR) spectrum of bare chitosan coatings and of the blends prepared at 25:75 ratios. Blends prepared at a 75:25 ratio also have these absorption bands, but with much smaller relative amplitudes probably due to the high-intensity peak at 1725 cm^–1^ corresponding to high amounts of PCL.

**Figure 1:**
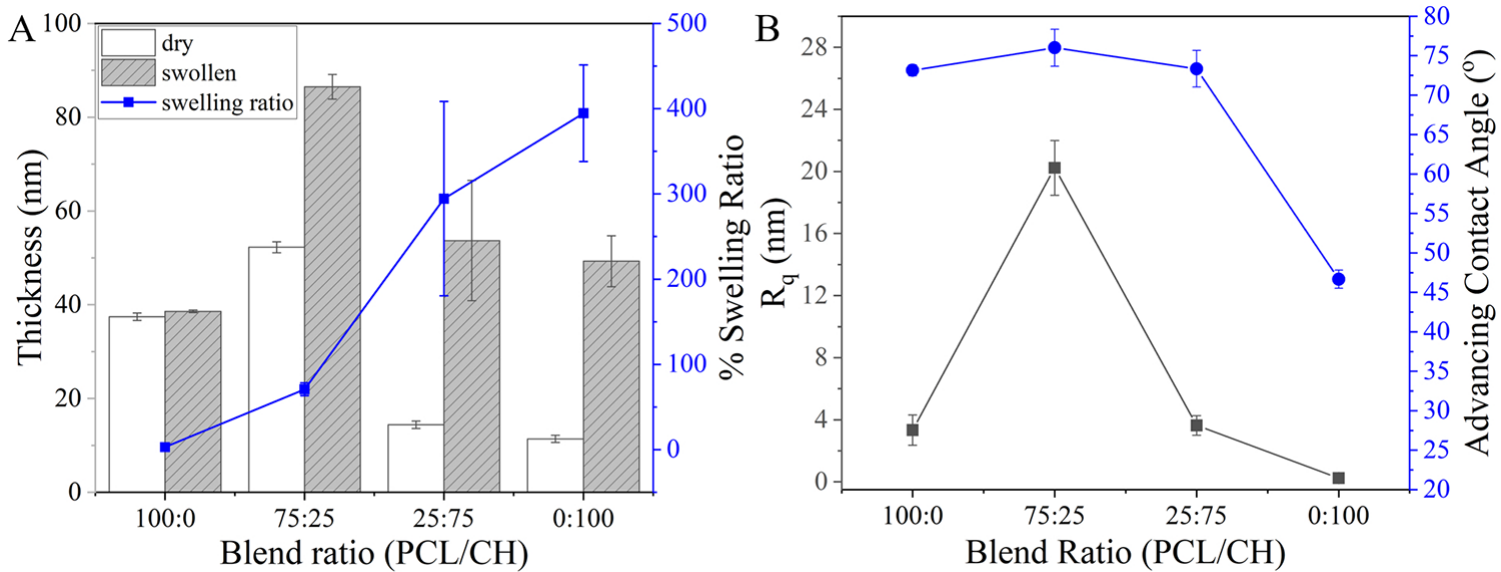
Measured dry and swollen thicknesses in 10 mM sodium phosphate buffer at pH 7.4 by spectroscopic ellipsometry (A), and AFM root-mean-square roughness *R*_q_ as well as advancing water contact angle (B) of the blend and homopolymer films.

For the thin films prepared on silicon wafers with native SiO_2_, the highest polymer concentration used in the 75:25 blend yielded the highest dry polymer thickness of around 55 nm. The lowest film thickness was measured for the pure chitosan films (*ca.* 10 nm). Polymer film thicknesses varied because in all films a constant chitosan amount was used for preparation to analyze fibrinogen and cell interaction with a fixed amount of hydrophilic polymer at the surface. The calculated swelling ratio of each film is displayed in Figure 1. It can be seen that PCL, being a hydrophobic polymer, did not swell much in the sodium phosphate buffer. The highest swelling degree was obtained for chitosan thin films, for which the swollen thickness is approx. 3 times higher than the dry thickness. Blend films have swelling ratios between the ones of pure polymer films, while a higher PCL amount (75:25) resulted in less swelling, as expected. Consequently, it is obvious that with increasing chitosan percentage within the films, the swelling ratio was also increasing due to the high water retention capability of chitosan.

For pure chitosan films, the advancing water contact angle was 47 ± 1° due to the high hydrophilicity of the chitosan. However, for pure PCL and blend films, the advancing water contact angles were similar around 73–75°, considerably higher than for the chitosan homopolymer film. From these results, a presence of the PCL at the film interface of the blend films can be assumed.

In addition, ζ-potential measurements performed at pH 7.4 led to a highly negative potential (−88 mV) for pure PCL films (Figure S2). The reason for this highly negative potential could stem from the preparation procedure. PCL was dissolved in an acetic acid solution, which can result in some residual acetate ions on the surface or lead to a breakage of some ester bonds, contributing to a negative charge of the surface.^40^ ζ-Potential measurements of the blends did not show a considerable difference (−32 and −38 mV for 75:25 and 25:75 PCL/CH blends at pH 7.4, respectively). For the pure chitosan film, a low negative potential (−15 mV) was detected (Figure S2). The chitosan ratio in the blend films increased the ζ-potential due to its isoelectric point (IEP) at pH 6.8, while PCL has an IEP of 3.2 (Figure S2). For both types of blend films, the IEP was similar at pH 5.7.

**Figure 2:**
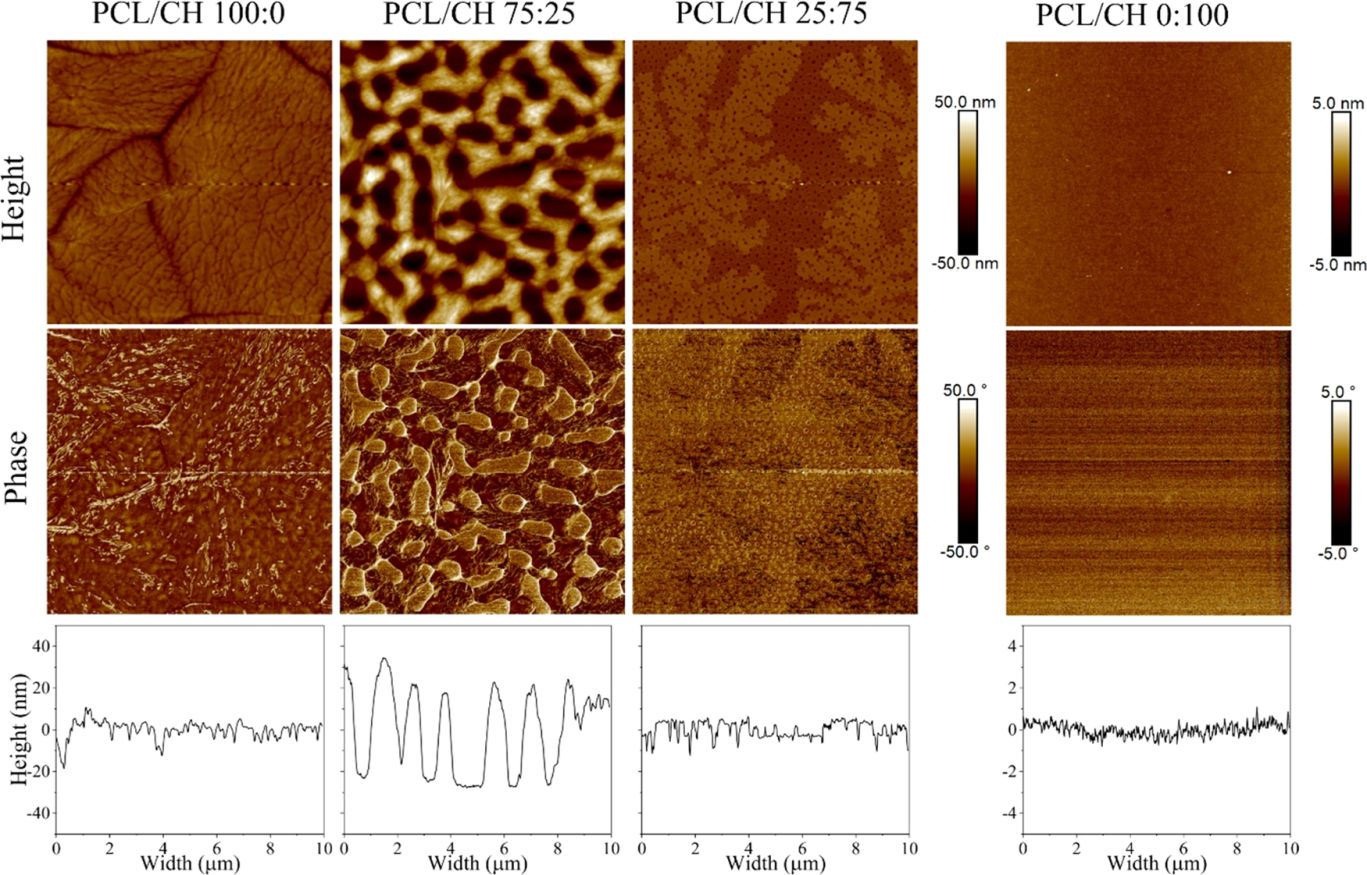
AFM height and phase images (10 × 10 μm^2^) as well as corresponding cross-sectional profiles of PCL/CH and their blend films. The *z*-scale is 50 nm for the height images except for the pure CH film, where it is 5 nm.

According to AFM images for a scanned area of 100 μm^2^ (Figure 2), it is apparent that the pure polymers produced morphologically homogeneous films, whereas the film surfaces of the blends showed visible domains. PCL is a semicrystalline polymer that forms spherulites during the spin-coating process.^41^ PCL tends to crystallize in clearly separated branched structures, a behavior that is often found for spin-coated blend polymer thin films.^42^ Furthermore, thin films prepared with pure polymers showed smoother surfaces (*i.e.*, lower *R*_q_ values) compared to the blends. Pure chitosan films had the lowest *R*_q_ value of less than 1 nm, while the *R*_q_ value for pure PCL films was 3.3 ± 0.9 nm. On the other hand, the roughness values for the blends prepared with 75:25 and 25:75 ratios were 20.2 ± 1.8 and 3.6 ± 0.6 nm, respectively.

Cross-sectional analyses of the surface profiles for a scanned area of 100 μm^2^ area were evaluated, as well (Figure 2 bottom). Thin films prepared with pure PCL (100:0), pure chitosan (0:100), and chitosan-rich blends (PCL/CH 25:75) have homogeneous profiles with differences between heights and valleys of less than 10 nm in general. Blends prepared with a 75:25 PCL/CH ratio show microdomains of (1.4 ± 0.4) μm size, which can be analyzed either from the phase or from the height image. Hence, a higher PCL amount in the blend resulted in a microdomain structure at the interface, which also leads to surfaces with the highest “peakiness” with an average distance between peaks and valleys of (49 ± 7) nm. This domain surface morphology is also reflected in the highest *R*_q_ values for the PCL-rich blend as displayed in Figure 1.

### Fibrinogen Adsorption

**Figure 3:**
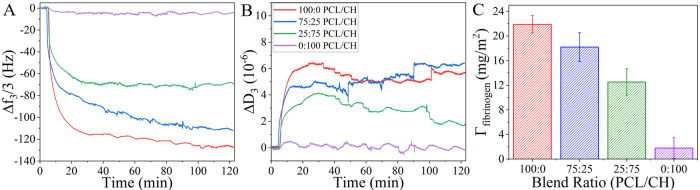
Typical QCM-D frequency (A) and dissipation (B) changes of fibrinogen adsorption onto PCL/CH and their blend films at 37 °C. Fibrinogen solution entered the chamber after 5 min and washing with PBS buffer started after 90 min of the experiment. Calculated Sauerbrey mass at the 120th min (C).

Protein adsorption has a significant influence on the *in vivo* performance of biomaterials because preadsorbed proteins may alter the succeeding cell response to the biomaterial surface. Therefore, after successful coating of the polymers onto silicon wafers and their film characterization, polymer films were prepared on silica-coated QCM-D crystals, and fibrinogen adsorption studies were conducted using QCM-D. Fibrinogen is an elongated extracellular protein, which is important for blood surface interactions as well as cell adhesion.^43,44^ A typical experimental run of the fibrinogen adsorption experiment is presented in Figure 3 based on frequency (Δ*f*_3_) and dissipation changes (Δ*D*_3_) *vs* time. Fibrinogen solution entered the chamber after 5 min, and washing with PBS buffer started after 90 min of the experiment. Looking at the fibrinogen adsorption on various polymer-coated silica sensor surfaces, the highest slopes in the frequency change (representing high adsorption rates) were observed in the first 10 min (Figure 3), followed by a decrease of the adsorption rate. One can generalize that most of the mass deposition was completed within 40 min of the adsorption experiment. Besides, there were no significant changes after washing with PBS buffer, which points to an irreversible binding of fibrinogen on the investigated polymer films.

From  in Figure 3, we conclude that fibrinogen adsorption does not affect the viscoelasticity of the polymer films of this study significantly.^20^ Therefore, we chose the Sauerbrey calculation for the adsorbed amount of fibrinogen. The highest adsorbed amount was monitored on pure PCL films. Moreover, it could be observed that with increasing content of chitosan in the blend films, the adsorbed amount of fibrinogen decreased. In accordance with this, on pure chitosan films, no significant protein adsorption was monitored.

It is known that roughness, hydrophilicity, and surface charge have an important effect on protein adsorption. While there is no pronounced difference in the surface roughness of pure PCL and chitosan films, PCL is more hydrophobic than chitosan, which was proven by contact angle measurements in this study (θ_PCL_^adv^ = 74 ± 1°, θ_Chitosan_^adv^ = 47 ± 1°). Proteins bind more strongly onto hydrophobic surfaces compared to hydrophilic ones^45^ because the contact of proteins with hydrophobic surfaces leads to changes in the protein conformation and to the release of water due to reorientation of hydrophobic amino acid residues at the interface.^42^ The induced protein unfolding can also lead to irreversible protein adsorption.^46^ The pure PCL film has an IEP of pH 3.2 (Figure S2) below the IEP of fibrinogen at pH 5.8.^47^ Thus, fibrinogen adsorbed to pure PCL films under electrostatically repulsive conditions, while it is known that protein adsorption also can take place under these conditions, in principle.^48,49^ Both effects, screening ions and hydrophobic forces that overcome the electrostatic interactions, could be the reasons for the increased protein adsorption onto the pure PCL film as discussed in other works.^42,50,51^ Surfaces with low or neutral charges are known to be more or less protein-resistant.^45^ Besides a small positive net charge of the surface (IEP of chitosan at pH 6.8; see Figure S2), the chitosan film is hydrophilic and has a high water retention capability, which leads to a water hydration layer at the polymer–solution interface.^3^ Proteins keep their native structure, and weak, mostly reversible protein adsorption occurs.^46,52^ As a consequence, fibrinogen does not adsorb significantly on pure chitosan films due to surface hydrophilicity, while the electrostatics are screened by the high ion content in the solution. So far, we have not discussed the influence of roughness on protein adsorption. When the surface roughness increases, an increase in the adsorbed amount due to the increase in the surface area was found in some studies until a certain roughness value,^53^ while higher roughness values may also reduce the adsorbed protein amount.^54,55^ In our study, roughness was the highest for the 75:25 PCL/CH films (*R*_q_ = 20.2 ± 1.8 nm) compared to a smaller roughness of pure PCL films (*R*_q_ = 3.3 ± 0.9 nm) in the range of the other films investigated. However, at the 75:25 PCL/CH blend surface, a smaller protein adsorbed amount is detected compared to the pure PCL film. No clear trend of fibrinogen adsorption on the surface roughness can be found. Since the blend contains hydrophilic chitosan, we conclude that the roughness had a minor effect in our adsorption studies, and adsorption was governed by the chitosan content.

### Cell Adhesion

The surface properties of a biomaterial have a significant effect on cell adhesion. Next to the study of adsorption of the model protein fibrinogen, QCM-D has been used to investigate the cell–polymer surface interactions in real time. Complementary studies were also performed in cell culture to verify cell adhesion and to obtain additional information about cell viability and morphological changes of the cells. In the course of the cell adhesion process, cells initially “sediment” to the surface with their spherical bodies. Then, cells “flatten” mostly by nonspecific interactions. When the appropriate proteins have secreted to the extracellular matrix (ECM), cells increase their surface contact area. “Cell attachment” continues *via* integrin-mediated specific interactions to form focal adhesion points, and subsequent “ECM remodeling” occurs. If the surface is suitable, cells “fully spread” with focal adhesion maturation and create stable contacts *via* actin skeleton reorganization to reach their maximum spreading area.^33,37,56^

**Figure 4:**
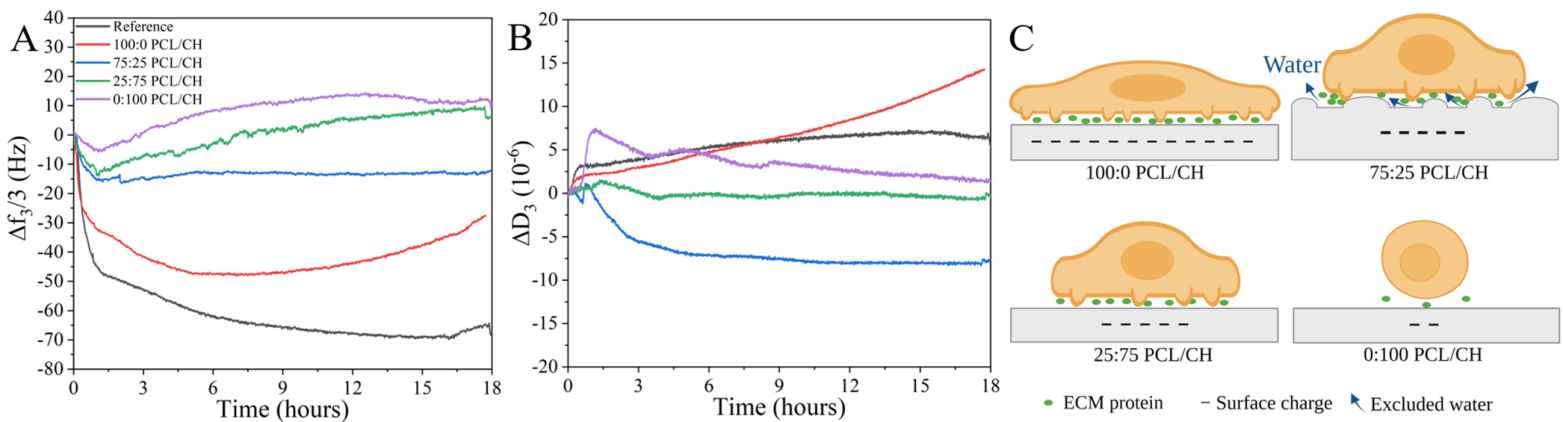
Representative frequency (A) and dissipation (B) changes during adhesion of hFOB cells on pure and blend PCL/CH films. The cell flow was stopped after 1 h. Frequency and dissipation data are shown for the 3rd overtone. Schematic representation of the hFOB cells and the underlying substrate (not to scale) (C). Corresponding ζ-potential measurements that underline the assumptions about surface charge in (C) can be found in the Supporting Information.

QCM-D experiments of cell adhesion were performed on uncoated silica reference sensors and polymer-coated surfaces (Figure 4). The QCM-D data are discussed once after one hour of adhesion to account for the cell sedimentation phase and after the complete experiment at 18 h. For the adhesion on silica, a decrease in the frequency signal (Δ*f*_3_ ≈ −45 Hz) accompanied by an increase in the dissipation (Δ*D*_3_ ≈ 3 × 10^–6^) was monitored throughout the first hour, accompanied by a small increase in dissipation (Δ*D*_3_ ≈ 3 × 10^–6^). These changes are attributed to the initial cell sedimentation onto the surface. Note that these signal changes stem from mass and viscoelasticity changes at the cell–surface interface where the actual adhesion occurs, with a maximum signal depth of the QCM-D method of around 250 nm.^18^ The mass of the whole cell is not detected by QCM-D due to its dimensions in the μm range. In the initial cell sedimentation stage, cells mostly interact by electrostatic nonspecific contacts.^57^ For adhesion at polymer-coated crystals, a similar trend in frequency change, but with a lower absolute change, was monitored, indicating smaller mass deposition/less cell adhesion as compared to the reference substrate. The highest frequency change (Δ*f*_3_ ≈ −30 Hz) for adhesion at the polymer films was monitored for pure PCL films. The negative groups on pure PCL are considered to act repulsive to the cell membrane at a long distance, but as the cells make intimate contact, the surface charge may polarize the biomolecules on the cell membrane and cause a strong binding.^58^ This phenomenon may explain the higher cell adhesion on pure PCL. Increasing the chitosan ratio in the blends resulted in less frequency change (*i.e.*, lesser initial cell sedimentation) after 1 h, which shows the surface dependence of the cell interaction (Figure 4A). The lowest Δ*f* was monitored on pure chitosan films (<5 Hz), which is in agreement with the very low fibrinogen adsorption on pure chitosan layers. The strong water hydration layer possibly made the initial nonspecific contact difficult between the cell membrane and the chitosan layer. Thus, cells could not easily form close contacts with the surface.

Dissipation changes after 1 h of adhesion at the polymer surfaces were as low as for the reference silica surface, except for the chitosan layer. For the chitosan film, there is a peak in Δ*D*, which decreases again when the pump was stopped after 1 h. Considering the softness of the underlying chitosan layer, this peak in dissipation could be attributed to viscoelastic properties of the interface, *e.g.*, transient mechanical disturbances of the chitosan layer due to the cell flow. This behavior cannot be attributed to the continued swelling of the pure chitosan layer as the signals were stabilized and normalized before the cell flow. Other contributions to the peak in Δ*D* could be the initial cell mass or mechanically trapped water at the cell membrane-–polymer interface. Although cell sedimentation increases the Δ*D* at all films, the frequency change for adhesion at the chitosan film is lower (*i.e.*, lower cell mass) than for the other polymer films at the end of 1 h. Furthermore, since the cells keep their spherical morphology during the initial sedimentation (the proximity between the surface and the ventral cell membrane is detected to be around 50 nm^59^), and the pure chitosan film has a very smooth surface (*R*_q_ < 1 nm), it is not likely for water to be mechanically trapped at the interface.

**Figure 5:**
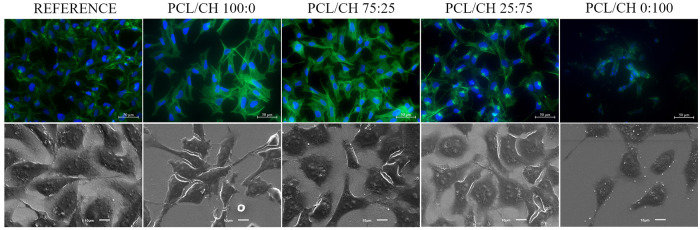
Morphology of hFOB cells grown 18 h on films with different PCL/CH ratios visualized by fluorescence microscopy (DAPI-stained nucleus and phalloidin-stained actin cytoskeleton) (top) and scanning electron microscopy (bottom).

When the pump was stopped after 1 h, on the reference silica surface, Δ*D* continued to increase and Δ*f* to decrease almost until the end of the experiment (Δ*f*_3_ ≈ −65 Hz, Δ*D*_3_ ≈ 5.3 × 10^–6^) under no-flow conditions. Since no mass was entering the measurement chamber after 1 h, changes in both signals are expected to originate from rearrangements at the cell–surface interface and/or altered cell–surface interactions rather than from further cell sedimentation.^59^ Further, considering general findings in other studies, we may conclude that these signal patterns can be related to “complete” cell spreading.^21,34,60−62^ In this direction, the decrease in Δ*f* monitored for the reference surface could be assigned to the increase in the surface contact area of the cells and adsorption of relevant secreted proteins. Additionally, the Δ*D* increase is more related to the cytoskeletal rearrangements and trapped water at the interface.^62,63^ Corresponding fluorescence images that were taken at the end of the cell adhesion process, *i.e.*, after 18 h, also verified that cells completely spread on the reference surfaces (Figure 5).

**Figure 6:**
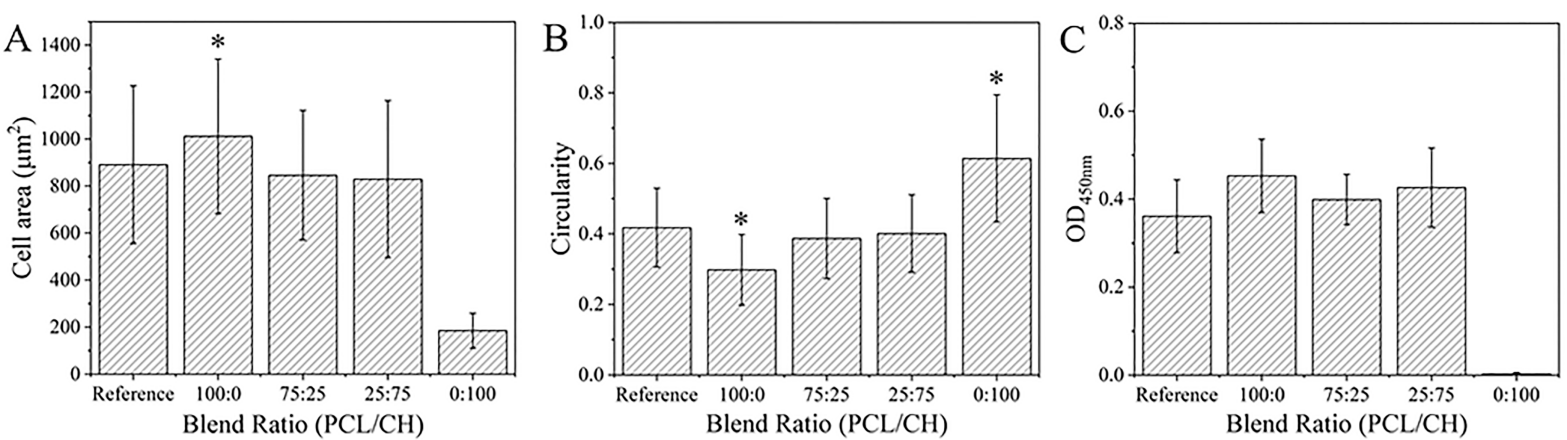
Measured hFOB cell area (A) and circularity (B) from fluorescence images; cell viability after 18 h; (C) statistically significant differences are seen with * for *p* < 0.05. * compared to results of blend films in (A) and to results of all films in (B).

For the polymer surfaces, changes in Δ*f* and Δ*D* differed from each other after 1 h in no-flow conditions. Particularly, the signal patterns measured for the pure PCL film were entirely different from the signals acquired for the other films (low Δ*f*, high Δ*D*). On the pure PCL film, the frequency drop continued for an additional few hours, but the dissipation continued increasing until the end of the experiment. The final Δ*f* change was smaller (*ca.* −30 Hz) as compared to the reference silica surface (*ca.* −65 Hz). As the surface contact areas were found to be similar for both surfaces in fluorescence image analysis, a smaller Δ*f* change could be considered as a smaller number of cells on the pure PCL film at first sight. However, cell viability assays indicated a similar number of viable cells after 18 h for the PCL and the silica reference surface (Figure 6). Thus, the smaller final Δ*f* cannot be attributed to cell detachment but to weaker cell–surface interactions compared to the reference silica surface. Image analysis revealed that cells grown on pure PCL films showed more elongated morphologies (*i.e.*, decreased circularity) compared to the cells grown on the reference surface (Figure 5). In general, hydrophobic surfaces with a contact angle >90° mostly hamper cell adhesion due to the underlying unfolded protein layer.^64^ For the PCL films prepared in this study, a relatively high negative ζ-potential was observed, which was discussed to lead to polarization of the cell membrane. Thus, cell adhesion and spreading are considered to be possible. Moreover, the reason for elongated morphologies could be the distributed charged (*i.e.*, carboxyl) groups on the pure PCL film that may push the cells to seek those regions instead of the hydrophobic groups. In this way, the cell membrane would extend and interact mostly with the charged groups. These cell behaviors may also cause the final Δ*D* to be higher for pure PCL (Δ*D*_3_ ≈ 13.5 × 10^–6^) compared to the reference silica surface (Δ*D*_3_ ≈ 5.3 × 10^–6^). We further state that the viscoelasticity per adhered mass (Δ*D*/Δ*f*), sometimes called the “acoustic ratio”, is noticeably higher for pure PCL. A higher acoustic ratio is associated with dynamic processes such as focal adhesion maturation and associated cytoskeletal changes,^62^ as they might occur when more elongated cell morphologies are formed.

The changes in Δ*f* and Δ*D* monitored for cell adhesion at the pure chitosan film in no-flow conditions show a different pattern (Figure 4). After the initial decrease in Δ*f*, the frequency change increased (*i.e.*, mass loss), even became positive until the end of the experiment. This can, on the one hand, be interpreted as continued cell detachment under no-flow conditions from the pure chitosan film. Complementary viability assays showed that there were no viable cells on the pure chitosan layer (taken after 18 h, Figure 6C), and adhered cells had an irregular shape (Figure 5). As pure chitosan films are highly resistant toward protein adsorption, probably the low surface charge of chitosan hindered cells from making further protein-mediated specific contacts with the surface, which are required for complete cell spreading. Thus, they started to detach from the surface. The trend of Δ*f* toward positive values indicates also other kinds of mass loss in addition to cell detachment, like stiffening of the highly swollen chitosan layer by a decrease of the water content with time. In our control experiments performed with a serum-free culture medium without cells, the signal was stable throughout the experiment, so we did not observe any positive trend in the Δ*f* signal (not shown), which eliminates the possibility of spontaneous stiffening of the pure chitosan layer. Therefore, we assume that the reason for the stiffening originates from the interactions of the cells with the pure chitosan surface. However, those interactions should be mostly nonspecific and weak. Because even though cells are present on the surface in the first few hours, the reduced cell viability and rounder morphologies observed at the end of 18 h indicated that the remaining viable cell number is small and also the interactions are weak. The increase in stiffness/rigidity could also be tracked from a decrease in Δ*D* with time. After a peak in Δ*D*, the dissipation gradually decreased with values close to zero after 18 h. This could be due to a decrease of the water content within the adlayer, as the cells detach from the soft chitosan film, but also because of rearrangements at the chitosan surface with time after the mechanical disturbances due to the cell flow.

A similar shape of the QCM-D signals was recorded for cell adhesion on chitosan-rich 25:75 PCL/CH blend films. Different from the pure chitosan, there were viable cells on the surfaces (Figure 6c) and the surface contact areas of the cells were similar to the cells grown on silica reference surfaces (Figure 6a). We concluded that there is no cell detachment based on the fluorescence microscopy images (Figure 5). Thus, we assumed that these adhered and spread cells should result in a drop in Δ*f*. However, an increasing Δ*f* under the no-flow condition was observed. Most likely, this could then be attributed to a decreased water content of the soft adsorbed film (stiffening) in time. Note that the swelling degree of 25:75 PCL/CH blend films is noticeably high. As discussed for the pure chitosan layer, this stiffening can occur as a result of the interactions of the cells on the surface. Similar to pure chitosan films, Δ*D* values returned to zero, indicating at decreased viscoelasticity during the cell adhesion process. Certainly, a decreased water content would stiffen the interface. Besides that, closer and more direct contacts of the cells to the 25:75 PCL/CH blend film require integrin-mediated specific interactions for the formation of focal adhesion/anchorage points, leading to reorganization of the local microenvironment (extracellular matrix remodeling). This remodeling causes the exclusion of some entrapped water between the cells and the surface throughout the spreading process replaced by appropriate excreted matrix proteins, all of which increase the rigidity of the interface.^17,59^ All of these factors might result in low Δ*D* values measured at the cell-25:75 PCL/CH blend interface. However, unlike for the chitosan film, this increase in rigidity with time was not pronounced. After reaching a maximum in Δ*D* at the end of cell flow at 1 h, the Δ*D* decreased to zero after 4 h and then was more or less constant until the end of the experiment.

If the surface properties are appropriate, after completion of ECM remodeling, cells pass to the next stage of spreading, including focal adhesion maturation and associated cytoskeletal rearrangements. “Complete spreading” reflects itself as an increase in Δ*D* and Δ*D*/Δ*f* (acoustic ratio), as discussed for adhesion on the silica reference and on pure PCL films. The low Δ*D* value observed for the 25:75 PCL/CH blend implies that spreading of the cells is weak. This is supported by fluorescence microscopy image analysis because the surface contact areas of the cells on these blend films were smaller than for the cells grown on pure PCL films. Besides, cells showed rounder morphologies. The surface properties of the blend (due to the presence of chitosan) might cause a lag time to the secretion of ECM proteins from the cells. Thus, cells might remain in the ECM remodeling stage. Yet, the lower ζ-potential (−38 mV) of 25:75 PCL/CH blend films might support the initial cell adhesion compared to the lack of adhesion on pure chitosan films.

QCM-D and fluorescence microscopy results of cell adhesion at the 75:25 PCL/CH blend films can be discussed in a similar way. Attachment of cells on the 75:25 PCL/CH blend films was confirmed, and the surface contact areas of the cells were similar to the ones of cells grown on silica reference surfaces (Figure 5). The increase in interfacial rigidity, as inferred from the QCM-D data, was more pronounced for 75:25 PCL/CH blends. The dissipation signal decreased toward more negative values after the stop of the cell flow and did not increase again until the end of the experiment. Since the swelling ratio of this blend was low compared to the chitosan-rich layers (Figure 1), it is difficult to assign this negative dissipation completely to layer stiffening. Furthermore, the ζ-potential values of the 75:25 PCL/CH blends do not differ significantly from the ζ-potential of chitosan-rich blends. From the AFM images, it can be seen that 75:25 PCL/CH blends exhibited a distinct topography with high surface roughness and a domain structure. The cavities of this relatively rough surface topography could contain much more water at the beginning of the cell adhesion process as compared to the other investigated surfaces. Even though the 75:25 PCL/CH film might not be as soft as compared to the pure chitosan film and the other blend films, the drastic decrease in Δ*D* could be the result of the replacement of a considerable amount of water from these cavities with excreted ECM proteins during the spreading process of the cells, decreasing the viscoelasticity of the cell–blend interface.

## Conclusions

QCM-D, especially when combined with complementary techniques, is shown to be highly useful to evaluate cell–surface interactions. However, the number of QCM-D studies investigating cell adhesion on polymeric surfaces is very limited. In this study, we used QCM-D to study the adhesion of hFOB cells on well-characterized homopolymer PCL, chitosan, and blend films in situ in real time. Thin films were prepared by spin-coating and then analyzed with respect to their physicochemical interface properties. Chemical analysis performed by ATR-FTIR spectroscopy showed that all of the polymers were successfully coated onto the substrates. Whereas the blend films showed visible domains, the homopolymer films were morphologically homogeneous and smoother. Dynamic contact angle measurements resulted in similar contact angles (*ca.* 73–75°) for PCL homopolymer and blend films. In contrast, chitosan homopolymer films were more hydrophilic (47 ± 1°). The highest swelling degree was obtained for chitosan thin films, in contrast to PCL films, which showed almost no swelling. The highest negative ζ-potential at pH 7.4 was measured for PCL homopolymer films and decreased with an increasing ratio of CH in the films. Fibrinogen adsorption was investigated, and a decreasing adsorbed amount of fibrinogen with increasing chitosan ratio was observed, which is due to the high hydrophilicity of the chitosan because electrostatic interactions were screened in the PBS buffer medium.

Cell adhesion was monitored for 18 h by QCM-D and resulted in different QCM-D signal patterns due to the differences in the interfacial properties. For the initial cell sedimentation stage, similar to the fibrinogen adhesion, a higher ratio of chitosan in the films led to a less negative frequency change (*i.e.*, less adhered cell mass). On chitosan homopolymer films, cells show low adherence, in general, as well as reduced viability and round cell morphologies as obtained by complementary cell culture assays. This finding was attributed to the low surface charge and high water retention capability of chitosan, which could hinder cells from making further protein-mediated specific contacts with the surface required for complete cell spreading. On PCL homopolymer films, hFOB cells completely spread and presented elongated morphologies, probably due to the polarization of the cell membrane by the highly charged PCL surface. In the QCM-D data, the highest frequency drop and the highest change in viscoelasticity per adhered mass (Δ*D*/Δ*f*) occurred upon cell adhesion at the PCL films. This points to dynamic processes during cell spreading at the PCL films such as focal adhesion maturation and associated cytoskeletal changes. On the blend films, a similar number of adhered cells with similar morphologies was detected compared to the reference surfaces. Better cell adhesion than on pure chitosan films is supported by lower ζ-potentials of the blend films. For soft 25:75 PCL/CH blend films, low Δ*D* values were interpreted as stiffening of the interface due to extracellular matrix remodeling and a decrease of the water content in time. This increase in rigidity was more pronounced for 75:25 PCL/CH blends. Since for the PCL-rich blend the swelling ratio was lower compared to the chitosan-rich blend, the decrease in ΔD was attributed to the replacement of water from the cavities of the highly rough 75:25 PCL/CH blend surface by extracellular matrix proteins. Contrary to the findings for PCL homopolymer films, we conclude from the rigidity of the cell–blend interfaces that cells remained in the ECM remodeling stage and could not pass on to the stage of spreading. Thus, we showed that polymeric biomaterial surfaces can be designed to tune cell interactions and to control stages of cell adhesion for specific application requirements.

## Materials and Methods

### Materials

Polycaprolactone (PCL) (440744), fibrinogen (FIB) (F8630), phosphate-buffered saline (PBS) tablets (P4417), fetal bovine serum (FBS) (F7524), 4-(2-hydroxyethyl)-1-piperazineethanesulfonic acid (HEPES), and trypan blue (TP154) were purchased from Sigma. Acetic acid was supplied from Merck. Chitosan (85/300/A1) was obtained from Biolog. Dulbecco’s modified Eagle’s medium (DMEM), trypsin/EDTA solution, and penicillin–streptomycin were obtained from Pan Biotech. Cell-counting kit-8 from Dojindo Molecular Technologies, 4′,6-diamidino-2- phenylindole dihydrochloride (DAPI; 100 nM; ≥97%) from Santa Cruz Biotechnology, Inc., and the phalloidin CruzFluor 488 conjugate were used.

### Methods

#### Preparation of Polymer Blends

For the preparations of the blends, a constant chitosan amount was used and mixed with a varying amount of PCL. A chitosan solution (1.5% in 0.5 M acetic acid) was mixed with a suitable amount of PCL solution (1 or 0.5% in glacial acetic acid) to reach 75:25 and 25:75 (v/v) blend ratios. In all mixtures, the acetic acid concentration was 80% for solubility reasons. Silicon wafers were cut in pieces of 1 × 2 cm^2^ and cleaned with absolute ethanol two times in an ultrasonic bath. Afterward, they were activated with an oxygen plasma (Plasma Cleaner PDC-002 with PlasmaFlo PDC-FMG-2, Harrick Plasma) for 1 min. Polymer mixtures (100 μL) were spin-coated at 4000 rpm with an acceleration rate of 2000 rpm/s for 1 minute (Spin150 spin coater, Polos, Putten, The Netherlands).

#### Surface Characterization

The ATR-FTIR spectra were obtained using an evacuated FTIR spectrometer Vertex 80v (Bruker, Ettlingen, Germany) equipped with a mercury–cadmium–telluride (MCT)-detector (InfraRed Associates Inc., Stuart (FL)) and a multiple reflection ATR-Si-wafer unit (Bruker, Ettlingen, Germany). The spectral range was 4000–1000 cm^–1^, and 500 scans were performed for each measurement.

The surface morphology of the films was characterized by atomic force microscopy AFM, Dimension 3100 (Digital Instruments, Inc., California), in the tapping mode using tips of the type BSTap (Budget Sensors, Bulgaria) with a resonance frequency of ∼75 kHz, a spring constant of 3–4 N/m, and a radius of ∼8 nm. All images were processed with Nanoscope software (Bruker, Germany), and roughness values were calculated as root mean square of the average of the profile heights (*R*_q_).

Dynamic water contact angles on the thin films were analyzed with an OCA20 contact angle device (DataPhysics Instruments GmbH, Filderstadt, Germany). Advancing water contact angles (θ_adv_) were determined from the dynamic dispensing and redispensing of water droplets (5 μL) with a flow rate of 0.5 μL/s. Analysis of the contact angles was performed with the needle embedded in the droplet. Contact angles were determined by the tangent of junction between the drop outline and the contact point of the solid surface and the sessile drop. All contact angle measurements were conducted at room temperature.

#### Film Thickness Measurements

By ellipsometry, the change of the polarization state of the light after reflection from a sample surface can be analyzed. Dry thicknesses of the thin films were modeled from ellipsometric data obtained with an α-SE ellipsometer (J. A. Woollam Co., Inc., Lincoln NE). A multilayer box model consisting of a silicon substrate, a silicon oxide layer, and the polymer layer was used. Optical constants of the silicon substrate and the SiO_2_ layer were taken from the database (CompleteEase software version 4.64, J. A. Woollam Co., Inc., Lincoln NE), and the optical dispersion of the polymer layer was modeled by a Cauchy function.

#### Swelling Experiments

*In situ* swelling experiments were performed at an α-SE (J. A. Woollam Co., Inc., Lincoln NE), using a batch cuvette with a fixed angle of incidence of 70° (TSL Spectrosil, Hellma, Muellheim, Germany). All measurements were performed with 10 mM sodium phosphate buffer at pH 7.4 in the spectral region of 370–900 nm. Analysis was done with CompleteEase software (version 4.64, J. A. Woollam Co., Inc., Lincoln NE).

To evaluate the swollen film thickness, a multilayer box model consisting of silicon, silicon dioxide, and a polymer layer was applied, as for the dry films, using a Cauchy dispersion to model the refractive index of the swollen polymer layer. The ambient refractive index *n*(λ) of the buffer solutions was measured with a digital multiple-wavelength refractometer DSR-lambda (Schmidt + Haensch GmbH Co., Berlin, Germany) at eight different wavelengths from 435.8 to 706.5 nm. The swelling degree of each polymer blend was calculated with eq 1. Dry polymer thickness (*d*_dry_) and swollen thickness are shown as *d*_wet_.
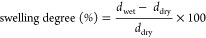
1

#### Fibrinogen Adsorption Studies

For the QCM-D studies, quartz crystal sensors (QSX 335) with an SiO_2_ surface were used. The SiO_2_ surface was activated with oxygen plasma (Plasma Cleaner PDC-002 with PlasmaFlo PDC-FMG-2, Harrick Plasma) for 1 min. After that, polymer solutions (100 μL) were spin-coated onto the sensors (Spin150 spin coater, Polos, Putten, Netherlands), and then, each sensor was mounted in the QCM-D flow chamber. The phosphate-buffered saline (PBS) solution was sent into the chamber with a velocity of 100 μL/min (IPC, Ismatec, Wertheim, Germany) until a stable baseline in frequency and dissipation for the 3rd to the 11th overtone was reached. The QCM-D cell was kept at 37 °C throughout the whole experiment. Then, a fibrinogen solution with a concentration of 0.25 mg/mL prepared in PBS at pH 7.4 was sent into the chamber. Fibrinogen adsorption was monitored for 90 min. After that, the buffer solution was sent into the chamber again to remove nonspecifically adsorbed proteins. The changes in frequency and dissipation were recorded, and the change in frequency and dissipation of the 3rd overtone was used for the interpretation of the results. Protein adsorption experiments were performed at least 3 times.

#### Cell Culture

For the cell adhesion studies, human fetal osteoblastic cells (hFOBs) were used. Cells were grown in tissue culture flasks in Dulbecco’s modified Eagle’s culture medium (DMEM) supplemented with 10% (v/v) fetal bovine serum (Sigma-Aldrich) and 1% (v/v) penicillin–streptomycin at 37 °C with 5% CO_2_. When the cells reached 80% confluency, they were detached from the cell culture plate by adding 0.25% trypsin–EDTA solution at 37 °C for 5 min. Trypsin was inactivated by addition of serum containing DMEM, and cells were collected by centrifugation at 1000*g* for 5 min. The pellet that contains cells was suspended with a fresh medium, and viable cells were determined after mixing the cells with a ratio of 1:1 with Trypan Blue; afterward, the cells were counted with a hemocytometer under a light microscope.

#### Cell Adhesion Studies by QCM-D

The cell adhesion experiments were performed in a flow module by QCM-D (QCM–Z500, KSV Instruments, Finland). QCM-D sensors were inserted into the instrument, and to reach the thermal equilibrium, a waiting time of 1 h was maintained. Since the QCM-D experiments cannot be performed in a CO_2_ incubator, HEPES (25 mM) was added to serum-free DMEM as a supplemental buffering agent and 1% (v/v) penicillin–streptomycin solution was added to prevent bacterial growth. Then, this solution was sent into the chamber with a speed of 100 μL/min until a stable baseline was reached. After the signals were stabilized, a defined number of cells (100.000 cells/mL) in a serum-free medium was passed through the chamber with a speed of 100 μL/min for 1 h (6 mL) to monitor cell adhesion. The pump was stopped 1 h later, and cells were left in no-flow conditions for 18 h. The cell adhesion experiments were performed at least three times at 37 °C.

#### Microscopic Analysis

The morphologies of the cells adhered on the thin-film-coated glass coverslips were evaluated using a general cell staining protocol. Cells to be evaluated with fluorescence microscopy were fixed with 4% (w/v) formaldehyde for 15 min. After washing with PBS 3 times, 0.5% Triton X-100 (v/v) in PBS (15 min) was used for permeabilization. After washing again with PBS 3 times, the cell nuclei were stained with 4′,6-diamidino-2- phenylindole dihydrochloride (DAPI; 100 nM) in PBS for 15 min. To stain filamentous actin (F-actin), cells were incubated with the Phalloidin CruzFluor 488 conjugate (Santa Cruz) for 20 min at room temperature. After washing with PBS 3 times, fluorescence images were captured using an inverted fluorescence microscope (Zeiss Axiovert). The images were processed with Zen 3.4 software (Blue Edition). Cells to be evaluated with scanning electron microscopy were fixed with 2.5% glutaraldehyde solution in 0.1 M Cacodylate buffer at pH 7.4 for 1 h and washed with the same buffer. After drying, the samples were coated with gold/palladium.

#### Cell Viability Assay

For the viability assay, a Dojindo Cell-Counting Kit-8 (CKK-8) was used. First, cells were seeded on round coverslips including the thin polymer film-coated ones (50.000 cells) and grown for 18 h at 37 °C with 5% CO_2_. After the media was removed, cells were washed with sterile filtered 0.01 M PBS at pH 7.4. Cell-grown coverslips were transferred to new well plates and incubated for 2 h after DMEM mixed with 10% CKK-8 was added. At the end of incubation, solutions were transferred to 96-well plates and the change in color was measured at 450 nm.

#### Cell Morphology and Statistical Analysis

The morphological parameters of the cells (area, circularity) were analyzed from the fluorescence images of the cells grown on coated glass surfaces after fixing for 18 h and were analyzed with image analysis software (ImageJ). At least 50 cells were analyzed for each type of coating. Statistical analysis was performed using Minitab with the two-sample *t*-test; *p* values <0.05 were considered statistically significant. The circularity of the cells was calculated from the cell area and perimeter values as obtained by ImageJ according to [4π(cell area)/(cell perimeter)^2^].^42^

## Supporting Information Available

ATR-IR spectra of PCL/CH and their blend films; and ζ-potentials of PCL, CH, and their blend films measured in 0.001 M KCl solution (PDF)
