# Supplementary material for: Monitoring Cell Adhesion on Polycaprolactone–Chitosan Films with Varying Blend Ratios by Quartz Crystal Microbalance with Dissipation
Source: ACS Omega. 2023 May 5;8(19):17017–27. doi: 10.1021/acsomega.3c01055 (PMC10193393; doi:10.1021/acsomega.3c01055)
Supplement: Supplementary file 1 — ao3c01055_si_001.pdf [file ao3c01055_si_001.pdf]

Supporting Information

Monitoring Cell Adhesion on Polycaprolactone-Chitosan Films with Varying Blend Ratio by Quartz Crystal Microbalance with Dissipation

Ayşe Buse Özdabak Sert[1], Eva Bittrich[2], Petra Uhlmann[2], Fatma Nese Kok[1¤], Abdulhalim Kılıç[1*]

[1]Department of Molecular Biology and Genetics, Istanbul Technical University, 34469 Istanbul, Turkey

[2]Leibniz-Institut für Polymerforschung Dresden e.V., 01069 Dresden, Germany

*Corresponding Author

¤Deceased on May 28th, 2022

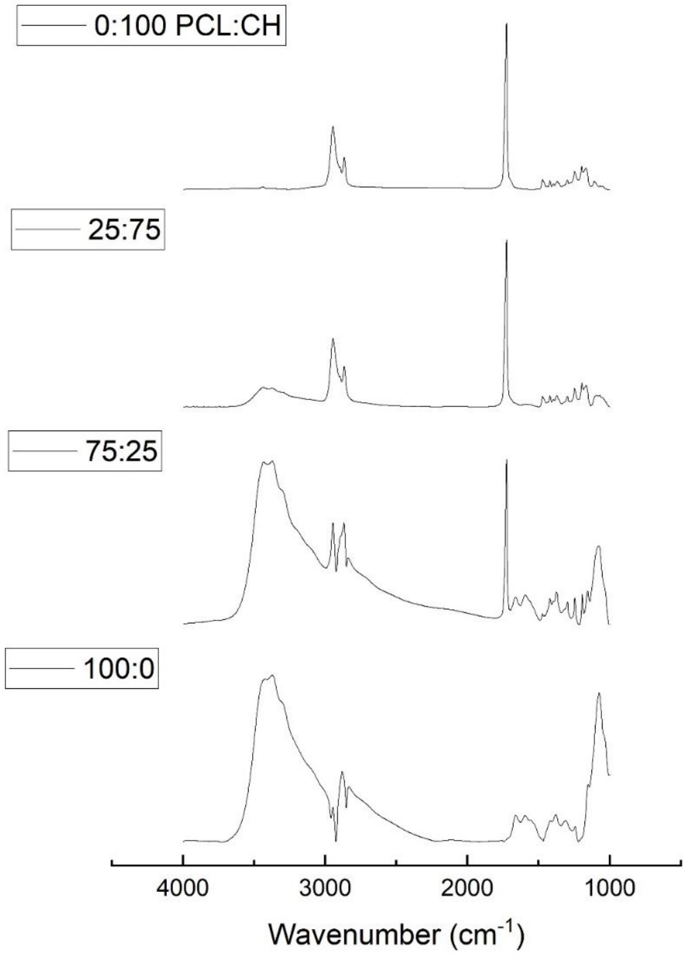

Figure S1: ATR-IR spectra of PCL:CH and their blend films.

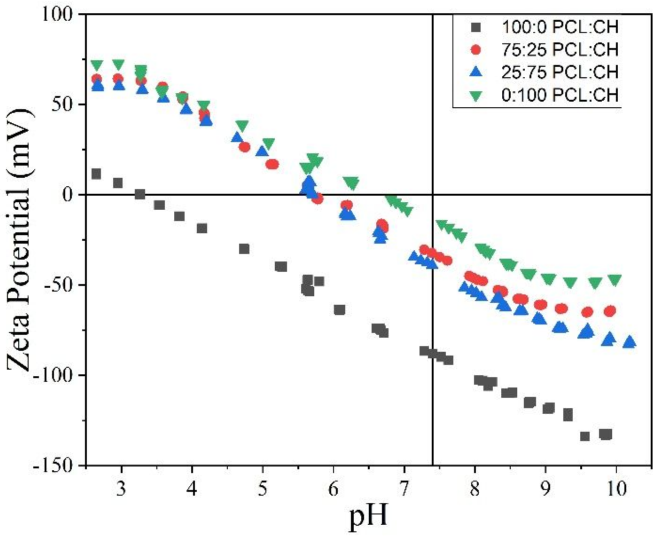

Figure S2: Zeta potential of PCL, CH and their blend films measured in 0.001M KCl solution.
